# Metronomic Chemotherapy with Vinorelbine Produces Clinical Benefit and Low Toxicity in Frail Elderly Patients Affected by Advanced Non-Small Cell Lung Cancer

**DOI:** 10.1155/2018/6278403

**Published:** 2018-08-27

**Authors:** Michela D'Ascanio, Aldo Pezzuto, Chiara Fiorentino, Bruno Sposato, Pierdonato Bruno, Alessio Grieco, Rita Mancini, Alberto Ricci

**Affiliations:** ^1^UOC Pneumologia, Hospital Sant'Andrea “Università Sapienza”, 00189 Rome, Italy; ^2^USL Grosseto, 58100 Grosseto, Italy; ^3^Sapienza University Department of Molecular and Clinical Medicine, Italy

## Abstract

**Background:**

Lung cancer is the leading cause of death worldwide. The treatment choice for advanced stage of lung cancer may depend on histotype, performance status (PS), age, and comorbidities. In the present study, we focused on the effect of metronomic vinorelbine treatment in elderly patients with advanced unresectable non-small cell lung cancer (NSCLC).

**Methods:**

From January 2016 to December 2016, 44 patients affected by non-small cell lung cancer referred to our oncology day hospital were progressively analyzed. The patients were treated with oral vinorelbine 30 mg x 3/wk or 40 mg x 3/wk meaning one day on and one day off. The patients were older than 60, stage IIIB or IV, ECOG PS ≥ 1, and have at least one important comorbidity (renal, hepatic, or cardiovascular disease). The schedule was based on ECOG-PS and comorbidities. The primary endpoint was progression-free survival (PFS). PFS was used to compare patients based on different scheduled dosage (30 or 40 mg x3/weekly) and age (more or less than 75 years old) as exploratory analysis. We also evaluated as secondary endpoint toxicity according to Common Toxicity Criteria Version 2.0.

**Results:**

Vinorelbine showed a good safety profile at different doses taken orally and was effective in controlling cancer progression. The median overall survival (OS) was 12 months. The disease control rate (DCR) achieved 63%. The median PFS was 9 months. A significant difference in PFS was detected comparing patients aged below with those over 75, and the HR value was 0.72 (p<0.05). Not significant was the difference between groups with different schedules.

**Conclusions:**

This study confirmed the safety profile of metronomic vinorelbine and its applicability for patients unfit for standard chemotherapies and adds the possibility of considering this type of schedule not only for very elderly patients.

## 1. Background

Lung cancer is the leading cause of cancer death worldwide. Tobacco is so far the most important cause of lung cancer, along with other associated risk factors such as air pollution and occupational exposure to hazardous chemicals [1]. The aforementioned could affect the response to chemotherapy agents [2]. Different histological patterns have changed over the last 25 years. Indeed, there has been an apparent increase in adenocarcinoma in both genders [3]. Over the last few years, advances in systemic treatment of lung cancer have been made, in particular in non-small cell lung cancer (NSCLC). However, treatment effectiveness in advanced stage IIIB or IV non-small cell lung cancer (NSCLC) without an EGFR-sensitizing mutation or ALK gene rearrangement has reached a plateau and it could depend on histological features, performance status (PS), age, and comorbidities.

The recommended therapy in patients with PS 0 to 1 consists of platinum-doublet therapy, whereas in patients with ECOG-PS 2, combination or single-agent chemotherapy or palliative care alone is being currently used [4].

The new frontier is the immunotherapy which encompasses several molecules that showed to be effective, such as nivolumab, atezolizumab, and pembrolizumab, demonstrating promising effects by allowing the immune cells to recognize cancer cells but limited by warnings and side effects [5].

A high proportion of lung cancer diagnosis occurs in elderly patients (≥ 65 years), who often represent the most challenging population to be treated, due to age-related disorders that increase the probability of drug-drug interactions and treatment-related toxicities [6].

Single-agent chemotherapy (vinorelbine, gemcitabine, and docetaxel) remains the recommended treatment for unfit or patient with comorbidities, who are more likely to develop treatment-related adverse events [3]. An unfit patient was considered a heterogeneity population based on some parameters such as stage, ECOG-PS, creatinine level, and smoking exposition, and they are not considered available for cisplatin-based chemotherapy [3, 7].

The term “metronomic therapy” refers to the continuous, often daily, administration of oral chemotherapy agent at fixed dosage, which aims to reduce toxicity and to prolong disease control.

Metronomic vinorelbine (mVNR) has been proposed as a possible alternative therapeutic regimen in patients with NSCLC [8]. Metronomic chemotherapy is based on the chronic administration of chemotherapeutic agents at low, minimally toxic doses and with no drug-free breaks.

Several clinical studies have pointed out the potential efficacy and low toxicity of metronomic chemotherapy with potential multitarget properties in cancer patients [9].

Due to its safety profile, treatment with metronomic VNR seems tailored for patients in whom the full-dose treatment is contraindicated or where the risk-benefit balance is not favorable, including elderly patients, patients with poor PS, and patients with important comorbidities [10–12].

The aim of the present study was to evaluate activity and safety of mVNR therapy in a sample of patients with advanced NSCLC stage IIIB or IV, poor performance status (PS≥ 1), and comorbidities (metabolic renal, hepatic, or cardiovascular disease). The exploratory analysis was done to compare different groups of patients according to the schedule of treatment and the effect of age in terms of progression-free survival (PFS).

## 2. Materials and Methods

### 2.1. Patients

In this study, we retrospectively analyzed 44 patients including elderly (over 60 years at the time of their NSCLC diagnosis) from our outpatients department (34 males, 10 females; median age 77 years] suffering from advanced NSCLC EGFR and ALK wild-type status (IV stage according to WHO classification system) and who were assigned to treatment with mVNR.

All patients showed an Eastern Cooperative Oncology Group ECOG-PS≥1 and at least 1 serious comorbidity such as metabolic renal, hepatic, and cardiovascular disease. Patients were either smokers or former smokers. Baseline demographic data are summarized in Table 1.

Thirty-four patients had histological diagnosis of adenocarcinoma, eight patients reported squamous cell carcinoma, and only two cases showed unspecified NSCLC (Table 1).

An ethical committee approval for this study was provided along with an informed consent through a resolution number 3382.

### 2.2. Scheduled mVNR Treatment

Thirty-eight patients received mVNR treatment after failure of conventional chemotherapy, while 6 patients received treatment as first-line therapy.

mVNR was administered orally. The therapy was administered at home and discontinued in presence of serious toxicity or disease progression.

Patients with ECOG-PS >2 or patients with two or more comorbidities underwent oral metronomic vinorelbine at 30 mg three times a week, whereas 40 mg three times weekly was used in patients with ECOG 1 and only one serious comorbidity.

Patients were reassessed every 2 weeks with clinical examination, complete blood cell count, and serum chemistry analysis. Radiologic response was evaluated with a whole body CT or CT / PET scan every 3 months from the first administration.

The primary endpoint was progression-free survival (PFS), defined as the relapsed time from treatment initiation to progression disease (PD) or death for any cause.

PFS was also used to compare patients based on different scheduled dosage (30 or 40 mg x 3/weekly) and age (more or less than 75 years old).

Toxicities were evaluated according to Common Toxicity Criteria Version 2.0. We classified adverse events into hematologic including anemia and leukopenia and nonhematological events (Table 2).

### 2.3. Statistical Analysis

All clinical data were assessed as mean values for continuous variables and as numbers and percentages for categorical variables. The PFS was analyzed with Kaplan Meier method; Log rank tests were used to detect the differences between groups. The statistical analyses were carried out by using GraphPad (version 5). The statistic significant value was set at p<0.05.

## 3. Results

The observational period of our study was 12 months, from January 2016 to December 2016; the patients under study underwent metronomic therapy with a high compliance rate (85%), which is defined as consuming over 75% of the medication prescribed.

Median age was 77 and the main histotype adenocarcinoma was present in 77% of all study population.

The smoking status was the following: former smokers 79%, never smokers 13%, and current smokers 6% (Table 1(a)).

The schedule dosage of mVNR was 40 x 3 a week or 30 x 3 depending on ECOG-PS.

Patients with PS 2 were 18, whereas 26 patients had PS 1. Patients aged >75 were 31, whereas 13 were patients aged between 60 and 75. Vinorelbine was used as first-line in only 6 patients, whereas 38 patients were treated as second line.

After 3 months of mVNR therapy, none of the patients showed a complete response; 12/44 patients (27.3%) showed a partial response as their objective response rate (ORR) with a CI from 18 to 36% (Table 1(a)). 16/44 (36%) of patients had a stable disease. The clinical benefit considered as disease control rate (DCR) was observed in 63% of patients. Progression disease was observed in 16/44 patients (36%). Overall, 15/44 patients achieved 12 months of observational time.

**Figure 1:**
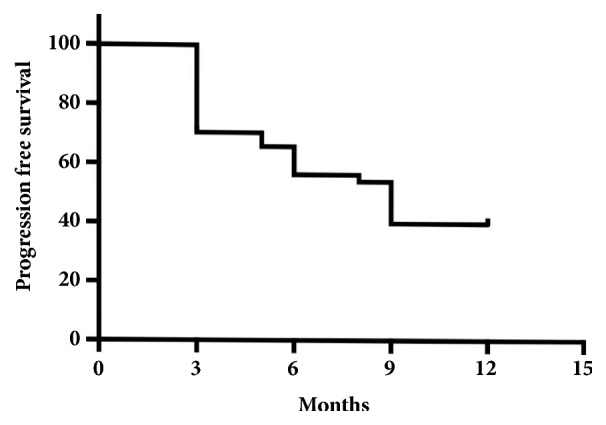
Kaplan Meier curve on global progression-free survival.

**Figure 2:**
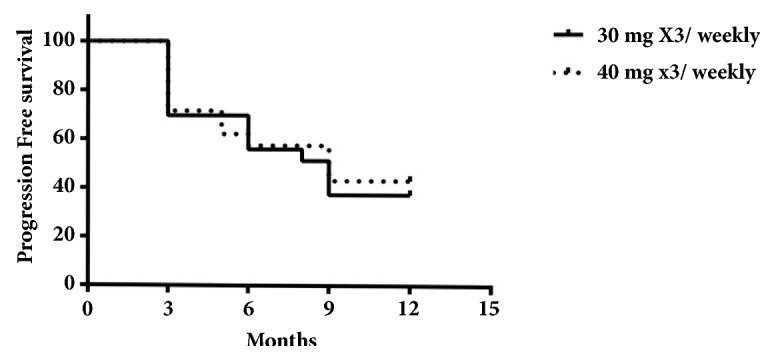
Kaplan Meier curve comparing the two types of schedule [30x3 versus 40x3 mgs/weekly].

**Figure 3:**
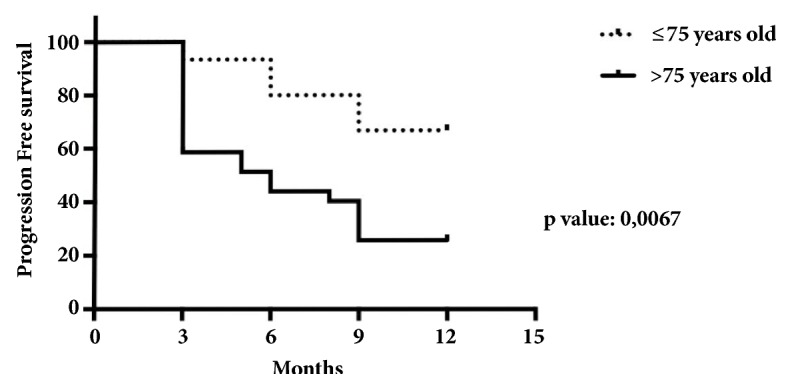
Kaplan Meier curve in function of age.

The mean PFS was 9 months (Figure 1, Table 1(b)) and median overall survival (OS) 12 months. No statistically significant results were found comparing the two types of schedule (30 versus 40mg x 3/weekly) with a HR 1.1 (Figure 2). Conversely, a statistically significant difference, in terms of PFS, was found referring to age (more or less than 75 years old) with HR 0.72 (p=0.006) (Figure 3, Table 1(b)).

Furthermore, our results demonstrated that single-agent vinorelbine administered three times a week was well tolerated and only 4% of the patients experienced grade 3 adverse reactions (neutropenia), thereby requiring granulocyte colony-stimulating factor administration. The nonhematological side effects included fatigue 25%, vomiting 4%, and diarrhea 11% (Table 2). No toxic deaths events occurred.

## 4. Discussion

Our data suggest safety profile of metronomic oral vinorelbine irrespective of schedule dosage. Thirty and 40 mgs three times a week were effective in the same way in terms of response rate.

Nevertheless, a difference regarding PFS was found in subgroups depending on age. In fact, the major benefit was observed in patients younger than 75yrs.

The disease control rate of 63% and the ORR suggest us to consider single-agent chemotherapy at metronomic schedule in patients affected by several comorbidities having a low ECOG- PS. We can use a low dosage since the clinical benefit was reached at both doses, 30 mg x 3/wk and 40 mg x 3/wk.

The OS, the ORR, and the mean PFS observed in our patients suggest that vinorelbine is effective in such subgroups of patients and that tumor cell growth was slowed by just one agent especially in elderly patients.

Several could be the targets of the aforementioned. mVNR efficacy seems to be related to the activity in tumor angiogenesis, being active on endothelial cells growth, on circulating tumor cells, and on endothelial progenitor cells in the bone marrow [13].

Although studies on metronomic treatment are restricted and have not been definitively accepted, several clinical trials have reported that metronomic is a well-tolerated treatment. Moreover, the real impact of metronomic chemotherapy on carcinogenesis and metastasis has not yet been clarified, as well as its potential as a sensitizer for radiotherapy. Its demonstrated therapeutic effects on tumor growth and its low toxicity allow us to consider this option in consolidation approaches in specific subsets of patients. Few data have also been reported on the effect of metronomic treatment in patients younger than 75yrs as we showed in the current study.

Chemotherapy resistance and failure are usually related to a group of tumor-initiating cancer stem cells (CSCs) involved in drug resistance, metastasis, and relapse of cancers. The use of chemotherapy at low doses alludes to the hypothesis that chemotherapeutical agents may act on different cell targets. Several lines of evidence indicate a significant antiangiogenetic activity together with a potential impact on the immune system [14, 15]. In addition, metronomic treatment seems to be able to prevent therapy-induced stromal activation with consequent activation of tumor-initiating cells in human desmoplastic cancers and orthotopic tumor xenografts [16]. Moreover, metronomic treatments may minimize the chemotherapy-host response that may counteract beneficial antitumor response [14].

Several studies point out that mVNR appears to show comparable effects with other chemotherapeutic agents, especially in elderly patients with a good safety profile [17].

Intravenously or orally administered vinorelbine demonstrated the same metabolism pattern, with identical pharmacokinetic-pharmacodynamics behaviour and it is able to maintain clinical efficacy as well [18].

In a randomized study carried out on elderly patients, oral vinorelbine administration (60 mg/m^2^) was compared to intravenous paclitaxel (90 mg/m^2^), where no significant differences in clinical benefit were observed [19].

Due to its safety profile [20], mVNR seems to be a suitable treatment also for patients in whom the full-dose treatment is contraindicated or when the risk-benefit balance is unfavorable, including not only elderly patients, but also subjects with poor PS and patients with important comorbidities [10–12].

In a Phase II study with mVNR administered three times a week in 43 naive patients, including unfit patients with a median age of 80 yrs, ECOG >2, ORR 18.6%, a median time to progression (TTP) of 5 months, and a median OS of 9 months were reported [21].

This study, as well as others based on a mathematical model, revealed that oral mVNR in elderly patients showed long-term disease stabilization and optimal patient compliance in elderly patients [22–25]. Furthermore, several lines of evidence demonstrated that vinorelbine improves survival in elderly patients and quality of life (QoL) [26]. Therefore, oral vinorelbine represents an appropriate agent for the treatment of selected elderly patients because of its good safety profile.

Vinorelbine could have multiple sites and mechanisms of action including the antiangiogenic properties and more recently an impact on the tumor microenvironment has been proposed as a potential effect involving the immune system [27]. We have to consider its multifunctionality since a large quantity of studies demonstrated that doublet chemotherapy as second-line therapy in elderly patients is more toxic and does not improve overall survival compared to single-agent [28].

A future application of vinorelbine may be considered in combination with other treatments, such as immunotherapy, since preclinical studies showed that vinorelbine could sensitize patients to immunotherapy through a synergistic action with PDL-1 receptors [29]. The use of single-agent in anticancer therapy along with best supportive care has often to be considered [30].

As a consequence, currently we should direct the choice of treatment according to the clinical evaluation of the patient, therefore tailoring the therapy.

We know from the literature that mathematical modelling may allow physicians to choose the treatment's regimen which encompasses the timing of administration, based on the evaluation of tolerability and pharmacokinetics [31]. Recent new trials reported that pharmacokinetics of the aforementioned vinorelbine is stable and the toxicity is associated with high dose of the drug [32]. Another recent prospective trial explored the activity and feasibility of mVNR 30 mg x 3/wk in selected groups of elderly patients, with advanced disease and poor prognosis owing to the presence of distant metastases and comorbidities. The trial reported a disease control rate of roughly 30%, higher in first-line treatment than in second-line with a PFS better in the first-line group [33].

Eventually, a potential synergism of action was demonstrated in combination with anti-EGFR in studies in vitro using lung cancer cell lines such as A 549, H-292. mVNR showed to be also effective in resistant cell clones by decreasing the expression of cyclin D1 and inhibiting the phosphorylation of AKT and ERK 1 which are proteins involved in cell proliferation[34].

## 5. Conclusion

Metronomic therapy appears to be an interesting area for the treatment of a specific subset of patients with low PS and comorbidities. Our data support this hypothesis. Moreover, they confirmed the good safety and activity profile of metronomic treatment irrespective of schedule dosage. Our results suggest that patients younger than 75yrs have a better response than older ones, underling the possible application of this treatment not only in very elderly patients, recommending the lowest schedule dose at 30mg x 3/wks in presence of significant comorbidities.

Therefore, metronomic oral vinorelbine could be the choice treatment for elderly patients unfit for traditional cisplatin-based chemotherapy, both in first- and second-line treatments because it is safe and well tolerated and is less expensive than other standard chemotherapies and more effective than best supportive care alone. It is important to take into account the fact that anticancer therapy is not able to totally eradicate cancer cells. The possible new paradigm for cancer therapy, in patients where full recovery from the disease is not possible, may be to reduce tumor burden over time. Indeed, our data are in line with this observation.

## Figures and Tables

**Table:** (a) Baseline study population characteristics [n = 44].

	Patients [n]	[%]
**Gender**		
Male	34/44	77%
Female	10/44	22%

**Median Age **[**range**]	77 [60-90]	

**Histology**		
Adenocarcinoma	34/44	77%
Squamous	8/44	18%
unspecified NSCLC	2/44	4%

**smoke status**		
Current smoker	3/44	6%
Former smoker	35/44	79%
never smoker	6/44	13%

**ECOG PS: 1**	24/44	54%
**ECOG PS ≥2 **	20/44	45%

**No. of chemothepy lines**		
**Line I chemotherapy**	6/44	13%
**Line II chemotherapy**	38/44	87%
**Response Rate**		
SD	16/44	36%
PR [partial response]	12/44	27%
PD	16/44	36%
Clinical benefit	28/44	63%
ORR	27% (CI 18-36%)	

**Table:** (b) PFS and OS

PFS mean [months]	9	
Overall survival median[months]	12	
PFS HR under 75/over 75 years	0.72	<0.05

**Table 2:** All grade treatment-related toxicities at final analysis [n = 44].

	All grade	Grade 3/4
**Non hematological toxicities**		
Fatigue	11/44 [25%]	0
Vomiting	2/44 [4%]	0
Diarrhea	5/44 [11%]	0
sensorial Neuropathy	1/44 [2%]	0
**Hematological toxicities**		
Anemia	12/44 [28%]	0
Leukopenia	10/44 [23%]	2/44 [4%]

## Data Availability

The retrospective data used to support the findings of this study are included within the article.
